# Mortality Among HIV Patients in ISRAEL: A 20-Year Retrospective Cohort

**DOI:** 10.3390/microorganisms14010118

**Published:** 2026-01-06

**Authors:** Daniel Elbirt, Mahmood Amer, Shira Rosenberg-Bezalel, Laliv Kadar, Shay Nemet, Ilan Asher, Ramon Cohen, Keren Mahlab-Guri

**Affiliations:** 1 Department of Allergy, Clinical Immunology and AIDS, Kaplan Medical Center, Rehovot 76100, Israelkerenmah@clalit.org.il (K.M.-G.); 2 Faculty of Medicine, Hebrew University of Jerusalem, Jerusalem 91120, Israel

**Keywords:** mortality rates, HIV, AIDS-related mortality

## Abstract

The objectives of our study were to determine the mortality rates, causes, and risk factors of people living with HIV in the modern antiretroviral therapy era, in a major HIV center in Israel. We retrospectively collected data from 1547 patients treated during 2001–2021. We used the Shapiro–Wilk test, Fisher’s exact test, Student’s *t* test, and chi-square to compare between patients who died and those who did not, and between patients who died from AIDS-related and non-AIDS-related causes. In total, 206 (13.3%) patients died. The causes of death were AIDS-defining diseases (33.5%), cardiovascular diseases (21.8%), non-AIDS infections (16%), and hepatic disorders (7%). The annual mortality rate was 1.31 ± 0.3%. Despite an increase in age (35 ± 13.2 in 2001, 49 ± 13.6 years in 2021; *p* < 0.001), the mortality rate decreased (2.12% during 2005–2008, 0.71% during 2018–2021; *p* = 0.0001). AIDS-defining diseases caused 75% of deaths during 2001–2002, and only 25% during 2019–2021. The proportion of cardiovascular deaths increased (8.3% in 2001–2003, 33.3% in 2019–2021; *p* < 0.001). Low CD4 and high viral load at diagnosis, male gender, non-MSM HIV acquisition (heterosexual transmission and people who inject drugs), and inability to achieve viral suppression because of non-compliance were risk factors for mortality. Mortality rates decreased during 2001–2021; however, the proportion of non-AIDS deaths increased. Early cardiovascular comorbidity screening and targeted adherence interventions in non-MSM populations and in patients with low CD4 are needed.

## 1. Introduction

Despite effective treatment, the HIV epidemic remains a global concern. According to the World Health Organization, there were an estimated 39.9 million [36.1–44.6 million] people living with HIV in December 2023; two thirds of them live in Africa. During the year 2023, 630,000 [480,000–880,000] people died from HIV-related causes and 1.3 million [1.0–1.7 million] people acquired HIV infection [1]. According to the Ministry of Health in Israel, 8831 people were known to be HIV-positive at the end of 2023 [2].

During the last decades, policies and guidelines regarding the management of HIV carriers changed dramatically [3,4,5]. While in the early period of antiretroviral drugs usage, the indication to begin treatment with combined antiretroviral therapy, in an asymptomatic HIV carrier, was a significant reduction in CD4 cell count to below 200 cells/µL or later to below 350 cells/µL; the common practice today is to start HIV medications as soon as possible, regardless of CD4 cell counts. In our center the “test and treat” policy (immediate ART initiation regardless of CD4 cell count) was adopted in 2013. The purpose of earlier initiation of treatment is to prevent deterioration of the immune system, to prevent non-AIDS complications such as heart disease and cancer (secondary to viral replication and inflammation) [6], and to reduce the rate of HIV transmission (epidemiological consideration) [7,8]. Safe, effective, and tolerable antiretroviral medications are now available and help to achieve good control of the disease [9]. Successful treatment with complete suppression of the HIV virus replication in the blood (viral load below the level of detection—currently 20 copies/mL), can be accomplished in most patients [10].

Successful antiretroviral treatment dramatically reduced mortality rates over the years [11]. A person living with HIV who receives antiretroviral therapy and is properly monitored can have normal life expectancy or very close to it [12]. A dramatic decline in AIDS mortality was observed in developed as well as in developing countries in the last two decades [13,14].

Although patients with successful antiretroviral treatment may not be immune deficient, they still have a chronic inflammatory state, which can be detected by a wide spectrum of pro-inflammatory markers [15,16]. The continuous inflammation, despite viral suppression, has an impact on morbidity, with an accelerated development of metabolic diseases, renal function impairment, osteoporosis, and cardiovascular complications [17]. Historical exposure to drugs with mitochondrial and metabolic toxicity such as Zidovudine (AZT), Stavudine (d4T), Lopinavir, Ritonavir, and others could also contribute to cardiovascular mortality.

Accordingly, the causes of death among people living with HIV have changed over the years. During the beginning of the AIDS epidemic the main causes of death were AIDS-related illnesses, including infections (opportunistic or non-opportunistic) and various tumors. In the recent years, death from non-AIDS-related tumors, liver disease, and metabolic and cardiovascular complications is on the rise [18,19].

In view of these facts, it is expected that mortality among people living with HIV in Israel will follow the global changes in mortality rates and causes.

The diversity of people living with HIV in our HIV center is unique. Most of our patients (about two thirds) are immigrants from Ethiopia with HIV subtype C and with mainly heterosexual transmission of HIV. These patients tend to present later for care and may differ in genetics or immunological responses. Other risk groups of our HIV patients are men who have sexual relations with men (MSM) (about 17%), people who inject drugs intravenously (about 8%), and mother to child transmission (about 5%).

In this study we characterized the mortality rates over the last 20 years, in one of the largest HIV centers in Israel, in view of this unique background and the risk factors, which are different from those described in other cohorts around the world [12,13,14].

## 2. Patients and Methods

Our HIV center, “Neve-or” in Kaplan medical hospital, is one of the largest HIV centers in Israel. We follow about 1700 people living with HIV (PLWH). We conducted a retrospective single-center study that included all of the patients who were treated at our HIV center during the years 2001–2021. We excluded patients with missing data regarding their CD4 and VL in the last year of the study, if they were alive according to the Israeli interior ministry of registration (lost to follow-up). We did not exclude patients who died, and we used their last available laboratory tests.

We collected demographic and clinical data, including sex, age at the time of HIV diagnosis and time of death, HIV risk group, duration of follow-up, treatment regimens (over the years), viral load (copies/mL), and immunological status (CD4 cells/µL) at diagnosis and before death (and also the date and cause of death) or at the end of the study period. AIDS was defined according to the CDC [20]. All patients were recommended to receive medical treatment according to the accepted clinical guidelines of the time.

Adherence to medications was assessed by the medical team, as good (above 90%) intermediate (60–90%), or poor (below 60%) according to medical history and documentation, HAART prescriptions compliance, and outpatient visits. This was a subjective evaluation in the absence of exact pharmacy refill data.

CD4 cell counts were measured using FACS—fluorescence-activated cell sorting—and presented as cells/μL [21], and HIV viral load is presented as copies/mL measured by RNA polymerase chain reaction, PCR [22].

## 3. Outcomes

We calculated and compared mortality rates and causes for each calendar year separately. Data regarding mortality (including those who were lost to follow-up) is automatically transferred to the medical records from the ministry of the interior. The cause of death was extracted from medical records (ICD-9/10 International statistical classification of diseases was used over the years). Two different investigators independently classified the causes as AIDS-related according to the Centers for Disease Control and Prevention revised classification system [20] and non-AIDS-related for all other causes to reduce bias.

We also compared mortality rates at our HIV center with those of the general Israeli population (according to the General Bureau of Statistics).

## 4. Statistics

Data are presented as means ± standard deviations. Continuous variables were tested for normality by Shapiro–Wilk test, and when abnormal distribution was found, non-parametric tests were performed. We used Fisher’s exact test, Student’s *t* test, and chi-square to compare demographic and clinical data with normal distribution between the study groups. *p* value < 0.05 was considered statistically significant. Data were analyzed using SPSS 25.

## 5. Results

In our study, we collected data of 1700 HIV-positive patients who were treated at our HIV/AIDS center during the years 2001–2021. We excluded 153 patients who were alive at the end of the study, but data regarding their CD4 or viral load was missing (lost to follow-up). The study included 1547 patients; 206 (13.3%) of them died during the years of the study.

The demographics and clinical characteristics of the patients who died during the years of the study compared to the patients who were alive at the end of the study period are presented in Table 1. Out of 206 patients who died, 135 (65.5%) were males compared to 762 out of 1341 (56.9%) of the patients who did not die, with a significant statistical difference (*p* = 0.036). The mean age at the time of HIV diagnosis was 40.81 ± 15.63 years for the patients who died compared to 33.02 ± 11.14 for the patients who were alive at the end of the study, with statistical significance (*p* = 0.0001). Most patients were immigrants from Ethiopia with heterosexual acquisition of HIV infection. This geographical risk group was more prevalent among the patients who died, 157/206 (76.2%), compared to the patients who did not, 85/1341 (68%); *p* = 0.006.

Regarding HIV parameters, the CD4 cell count in the peripheral blood at the time of HIV diagnosis was 214 ± 184 cells/μL in the group of patients who died compared to 296 ± 206 cells/μL in the patients who were alive at the end of the study (*p* = 0.0001). More than one third (37%) of the patients who died had a CD4 cell count below 200 cells/μL at the time of HIV diagnosis, but only 10% of those patients had an AIDS-defining illness at the time of HIV diagnosis (according to the CDC definitions [20]). Patients who died had significantly higher viral loads at the time of HIV diagnosis compared to the patients who were alive at the end of the study (Table 1). About 70% of the patients who died had a viral load greater than 100,000 copies/mL at the time of HIV diagnosis.

According to the clinical guidelines throughout the study period, highly active antiretroviral therapy (HAART) was recommended for most patients (84%) depending on their immunological status. As is shown in Table 2, about 45% of the patients who died were recommended to receive protease inhibitor (PI)-based therapy; nearly one quarter were recommended to receive non-nucleotide reverse transcriptase inhibitor (NNRTI)-based therapy, and approximately 30% were recommended to receive integrase strand transfer inhibitors (INSTIs). The adherence of the patients to medications was assessed as good by the medical team (according to medical history and documentation, HAART prescriptions compliance, and outpatient visits) in only 47% (Table 2). The immunological status of the patients who died did not improve significantly during the years of follow-up. The mean CD4 cell count at the time of death was 212 ± 203 cells/μL, similar to cell counts at the time of HIV diagnosis (*p* = 0.714). About 43% of the patients had a CD4 cell count of less than 200 cells/μL at the time of death compared to 37% at the time of HIV diagnosis (*p* = 0.34). Only 31% of the patients who died achieved undetectable viral load in at least one of the two consecutive HIV viral load tests before their death (laboratory tests were usually taken every 3 months during the years of the study) compared to 89.1% of the patients who were alive at the end of the study (*p* = 0.0001).

As expected, the mean follow-up time was shorter for the patients who died (9.31 ± 5.81 vs. 15.7 ± 6.4; *p* = 0.0001). The mean age at the time of death was 49.17 ± 15.64 years.

Overall 69 patients out of 206 (33.5%) died because of AIDS-related diseases, and 122 (59.2%) died from a non-AIDS-related condition. The cause of death was unknown for 15 patients (7.3%). As is shown in Table 3, the two groups had a similar male to female ratio and similar risk factors for HIV acquisition. People who inject drugs had higher non-AIDS mortality, but without statistical significance (*p* = 0.08). As was expected, diagnosis of HIV at older age (43.9 ± 16.4 compared with 36.6 ± 13) and death at older age (52.7 ± 16.2 vs. 43.9 ± 13.03) were significantly associated with non-AIDS-related mortality compared to mortality from an AIDS-defining condition (*p* = 0.003 and *p* = 0.0004, respectively). Low CD4 cell counts and high viral load at the time of HIV diagnosis were associated with both AIDS and non-AIDS mortality without a significant difference. As expected, a lower percentage of patients who died from AIDS-related diseases achieved complete viral load suppression before death (8.7% vs. 41.8%; *p* = 0.0001).

On average, during the study period, the yearly mortality rate was 1.31 ± 0.3% (range 0.53–2.17%). During the years of the study, the annual mortality rates decreased significantly. In 2001–2003, the annual mortality rate was 2.09%, compared to 0.97% in 2019–2021 (*p* = 0.0001). The decline in annual mortality occurred despite the increasing age of our patients (the mean age was 35 ± 13.2 years in 2001 and 49 ± 13.6 years in 2021). The mean age of the patients who died was 32 ± 19.2 in 2001 and 51 ± 11.8 in 2021.

**Figure 1:**
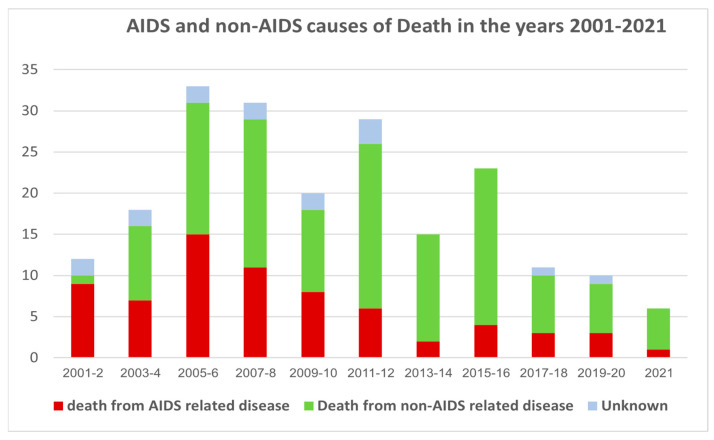
AIDS and non-AIDS causes of death in the years 2001–2021. The Y axis is the absolute number of deaths. The increase in the absolute number of deaths during 2006–2008 is related to the growing numbers of patients treated in our HIV center. During 2001–2002, AIDS-related diseases were 75% of the causes of deaths. During the last year AIDS-related disease was the cause of death in only 16.66%.

During the entire period of the study, 69 patients (33.5%) died from AIDS-related conditions. Over the years (as can be seen in Figure 1), the rate of patients who died from AIDS decreased. Thus, in 2001–2002 most deaths were due to AIDS-related conditions 9/12 (75%), while in 2021 AIDS-related deaths accounted for only 1/6 (17%).

On the other hand, 122 (59.2%) patients died from non-AIDS-related causes. The proportion of patients who died from a non-AIDS-related condition increased over the years from 8.3% in 2001–2003 to 90% in 2020–2021; *p* = 0.0001. This was especially prominent when we compared mortality from tuberculosis (as an AIDS-related cause) to mortality from cardiovascular disease (as a non-AIDS-related cause). In the first three years of study (2001–2003), there were only two deaths from cardiovascular disease out of 23 deaths (8.7%) and in the last three years of study (2019–2021), 5 of the 15 deaths in these years (33.3%) were attributed to cardiovascular disease (*p* = 0.09). Death from tuberculosis accounted for 8.7% of deaths in 2001–2003 and none of the deaths in 2019–2021 (*p* = 0.5).

**Figure 2:**
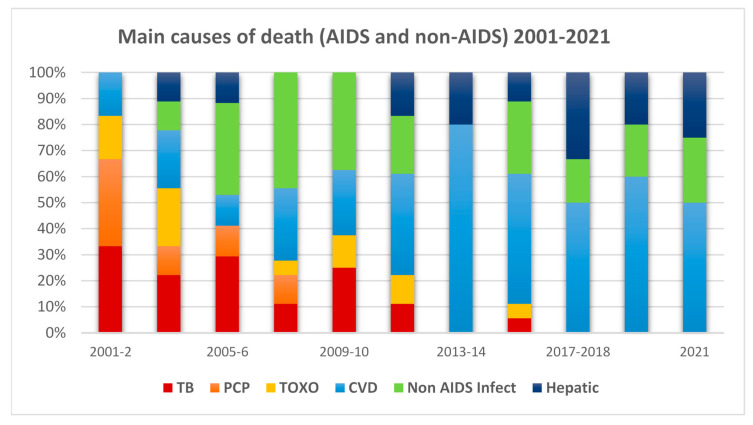
Main causes of death during the years of the study of AIDS-related diseases (TB—tuberculosis, PCP—pneumocystis pneumonia and TOXO—toxoplasmosis) and non-AIDS-related (CVD—cardiovascular disease, non-AIDS infections, and hepatic diseases).

The main three causes of death associated with AIDS (TB, PCP, and toxoplasmosis) and the major three non-AIDS causes of death (CVD, non-AIDS infection, hepatic disease) over the years are graphically described in Figure 2. Tuberculosis (TB) was the cause of death in 17 (8.3%) cases, pneumocystis Jiroveci pneumonia (PCP) in 9 (4.4%), cases and toxoplasmosis in 8 (3.9%) cases. Other causes of AIDS-related death were Mycobacterium Avium Complex (MAC) infection, Cryptococcal meningoencephalitis, Cytomegalovirus (CMV) infection, Candida infection, and Cryptosporidium infection, as well as AIDS-defining tumors such as lymphoma, Kaposi sarcoma, and cervical cancer. The main causes of non-AIDS-related deaths were cardiovascular disease in 46 patients (22.3%) and non-AIDS infection in 39 (18.9%) patients (severe pneumonia, Gram-negative bacteremia). Although the last two years of our study were during the COVID-19 pandemic, none of our patients died from COVID-19 infection. The third most common cause of non-AIDS deaths was liver disease in 15 cases (7.3%). Other causes of non-AIDS mortality included suicide, kidney disease, trauma, parasitic infections, violence, drug overdose, and non-AIDS-related cancers (lung cancer, esophageal cancer, gallbladder cancer, and liver cancer).

## 6. Discussion

The changes that have taken place in recent years in the treatment of HIV carriers are striking. Patients who are HIV-positive and who were sentenced to death in the early 1980s are now receiving effective antiretroviral therapy, with an excellent safety profile and few side effects and are expected to have an average or close to average life expectancy [3]. Over the years the mortality of HIV patients declined dramatically, along with changes in mortality causes [14]. AIDS-related mortality is slowly replaced by non-AIDS-related mortality [23].

In this study, we conducted a retrospective review of the deaths in the AIDS clinic of Kaplan Medical Center between 2001 and 2021. The study population was heterogeneous with both men and women and three different risk groups: geographic (mainly immigrants from Ethiopia), men who have sex with men (MSM), and people who inject drugs. During the years of the study, effective antiretroviral medications became the standard of care. During 2001–2010 modern antiretroviral therapy included a combination of nucleoside reverse transcriptase inhibitors with non-nucleoside reverse transcriptase inhibitors or with protease inhibitors. Later regimens were based on a combination of nucleoside reverse transcriptase inhibitors and integrase strand transfer inhibitors. These changes in therapy along with the emergence of single-tablet regimens had a major impact on the toxicity of medications, compliance of our patients, and eventually on mortality rates over time.

During the study period, 206 patients, 13.3% of all patients, died. The mean annual mortality rate was 1.23% per year, similar to mortality rates reported in other developed countries around the world [23]. Despite an increase of almost 14 years in the mean age of our patients, mortality rates declined by 66.5% from 2.12% in 2005–2008 to 0.71% in 2018–2021 (*p* = 0.0001). This decline reflects continuous improvement in the diagnosis and treatment of HIV patients with modern combinations of antiretroviral medications. A similar decline in mortality rates was observed by the ministry of health regarding the entire HIV population in Israel. The mortality rate of HIV patients declined from 2.03% in 2001 to 1.2% in 2012 and 0.86% in 2021 [2]. Globally, the mortality rates of people living with HIV were reduced by 46% from 2010 to 2021, by 33% in high income countries, and by 57.3% in sub-Saharan Africa [24].

Despite medical team recommendation and free access to effective antiretroviral therapy, about 34% died from AIDS-related disease (mainly tuberculosis, PCP, and toxoplasma). About 16.5% (34) of the patients who died, died in the first year after HIV diagnosis, Thus, late presentation is still an important cause of death. However, looking at the causes of death over time, it was revealed that the death from AIDS-defining diseases declined while the proportion of deaths from non-AIDS-related disease, such as cardiovascular disease, non-AIDS-related infections, and liver disease increased (Figure 1).

Figure 2 shows how the prevalence and proportions of death due to AIDS-defining conditions (tuberculosis, PCP, and toxoplasmosis) decreased over the years, while the proportion of mortality from non-AIDS-related conditions (cardiovascular disease, non-AIDS infections, and liver disease) increased. This is similar to other cohorts such as the Swiss cohort [23].

Despite the decline in mortality rates over the years, the mortality rate of people living with HIV is still higher compared to the general population in Israel (0.59% in 2005–2008 and 0.46% in 2018–2020) [25]. There are also differences regarding the causes of deaths. According to the Central Bureau of Statistics [25], the three most common causes of death among the general population in Israel are (in descending order) cancerous tumors, cardiovascular disease, and diabetes during all the years except for 2020, in which COVID-19 was the second cause of death. According to our study, cardiovascular diseases are now the leading cause of death among people living with HIV treated in our HIV clinic, while non-AIDS-defining infections and liver diseases come next.

In an attempt to characterize HIV-positive patients at increased risk for death, we compared the patients who died to the patients who were alive at the end of the study period. Male sex and older age at the time of HIV diagnosis were significantly associated with death, perhaps because these are established risk factors for cardiovascular diseases. Data regarding other cardiovascular risk factors such as smoking status and hyperlipidemia were missing.

Geographical risk factor for HIV acquisition (new immigrants from Ethiopia) was also associated with higher mortality, probably because of cultural and socioeconomic differences, language barriers, and stigma leading to lower compliance with therapy [26]. HIV subtype C, which is the most common subtype among Ethiopian HIV patients in our center, can also lead to greater disease progression compared to other subtypes [27].

As was expected, lower CD4, higher viral load at the time of HIV diagnosis, and shorter duration of follow-up were also associated with mortality, with statistical significance. These factors did not reach statistical significance when we compared AIDS-related deaths to non-AIDS-related deaths. Low CD4 and high viral load are associated with immunosuppression and AIDS, but viral replication can also accelerate atherosclerosis [6] and non-AIDS mortality.

The significant rise in the cardiovascular proportion of mortality from 8.7% to 33.3% during the years was despite routine assessment and treatment of our patients for classical cardiovascular risk factors (hypertension, diabetes, hyperlipidemia, and smoking status). High rates of cardiovascular disease and death (around 15%) among PLWH was also observed in other studies [28,29]. Changes in treatment regimens over the years could have confounded mortality trends by reducing AIDS mortality but increasing cardiovascular risk [30].

Cancer-related (non-AIDS-related malignancies) mortality was low in our study 4/191 (2.09%) compared to other studies (around 10%) [31]. Underreporting, lack of cancer screening in young patients for non-HIV-defining cancers, or competing mortality risks may explain those findings. However, like the trend in other countries [32], a rise in non-AIDS-related malignancies as the cause of death was observed during the years as only one case was the cause of death during the first 15 years of the study and three cancers were the cause of deaths in the last 6 years of the study.

The most important limitation of our study is the potential of selection bias since we excluded 153 patients with missing data regarding their CD4 and viral loads, who were lost to follow-up. However, information about death in Israel is presented in the electronic files, and we did not detect cases of mortality among those excluded (although some patients may have left the country). Another important limitation is the retrospective nature of our data collection with lack of lifestyle variables (especially smoking status), CD4 nadir, and others, precluding causal inference. Another limitation is the fact that estimation of compliance was according to clinical impression of the medical team, without pharmacy refill data. Another limitation is the missing data regarding the causes of deaths in 15 patients, which could have significant influence on the assessment of changes in mortality causes over the years. Cancer mortality, which is strikingly low compared to international cohorts may be a result of underdiagnosis or bias, since we did not cross-link deaths with the National Cancer Registry. Multivariant logistic regression to verify risk factors for death was not conducted.

Despite these limitations, our study is a long follow-up (20 years), single-center cohort with homogeneous care protocols and a diverse population not limited to a Western MSM cohort.

In conclusion, mortality rates decreased significantly during the years of the study (from 2.12% during 2005–2008 to 0.71% during the years 2018–2021; *p* = 0.0001). In addition, there has been a significant shift in the causes of death from AIDS-related causes (such as tuberculosis and PCP) to causes unrelated to AIDS (e.g., cardiovascular disease and non-AIDS-related infections). Despite these changes, mortality rates are still higher among people living with HIV, and the causes of deaths are still different from the causes of deaths of the general population in Israel.

Our findings support the integration of aggressive cardiovascular risk management and smoking cessation programs into routine HIV care, particularly for the ageing population and non-MSM groups. Further studies of mortality over the years in HIV centers are needed to further characterize risk factors for death and the need for action.

## Figures and Tables

**Table 1:** Characteristics of the patients who died during the study period compared to the patients who were alive at the end of the study period.

Demographic and Clinical Characteristics		Patients Who Died	Patients Alive at the End of the Study	*p*-Value
Number of patients		206	1341	----
Males		135 (65.5%)	762 (56.8%)	0.02
Females		71 (34.5%)	579 (43.1%)	0.02
Mean age at the time of HIV diagnosis (years)		40.81 ± 15.63 (0.1–81)	33.02 ± 11.14 (0.1–81)	0.0001
Risk group	Geographical with heterosexual transmission	157 (76.2%)	895 (66.7%)	0.006
	MSM	27 (13.1%)	251 (18.7%)	0.055
	People who inject drugs	18 (8.7%)	105 (7.8%)	0.67
	MTCT	3 (1.4%)	74 (5.5%)	0.009
	Other	1 (0.5%)	16 (1.2%)	0.71
CD4 at the time of HIV diagnosis (cells/µL)		214 ± 184 (4–873)	296 ± 206	0.0001
VL > 500,000 copies/mL at the time of HIV diagnosis		54 (26.2%)	123 (9.2%)	0.0001
Mean follow-up time (years)		9.31 ± 5.81 (0.1–26)	16.6 ± 6.4 (0.1–28)	0.0001
VL below detection limit ^1^ (in at least one of the two last tests)		63 (31%)	1196 (89.1%)	0.0001

^1^ Viral load < 400 copies/mL until 2007, viral load < 40 until 2010, viral load < 20 since 2011; MSM = Men who have sex with men, MTCT = Mother to child transmission, VL = Viral load.

**Table 2:** Other clinical details of the patients who died during the study course.

Demographic and Clinical Characteristics		Patients Who Died
Number of patients who died		206
Mean age at the time of HIV diagnosis in years (range)		40.81 ± 15.63 (0.1–81)
CD4 cell count at time of HIV diagnosis	Mean (range)	214 ± 184 (4–873)
	<200 cells/µL	76 (36.9%)
AIDS ^1^ at the time of HIV diagnosis		21 (10.2%)
Initial HAART class recommendation	PI based	76 (43.9%)
	NNRTI-based	38 (21.9%)
	Integrase-based	56 (32.3%)
	Other	3 (1.73%)
Adherence ^2^	Good	81 (46.8%)
	Intermediate	36 (20.8%)
	Poor	56 (32.3%)
Mean age at time of death in years (range)		49.17 ± 15.64 (1–91)
CD4 cell count close to the time of death	Mean (range)	212 ± 203 (1–1102)
	<200 cells/µL	88 (43%)
VL below detection limit ^3^ (in at least one of the two last tests)		63 (31%)
Death caused by AIDS-defining condition ^1^		69 (33.5%)

^1^ AIDS defined according to CDC definitions [2]; ^2^ Adherence as estimated by the clinic personnel during the visits. ^3^ Viral load < 400 copies/mL until 2007, viral load < 40 until 2010, viral load < 20 since 2011; HAART = Highly active antiretroviral therapy, PI = Protease inhibitor, Integrase = Integrase inhibitors, NNRTI = Non-nucleotide reverse transcriptase inhibitor.

**Table 3:** Clinical and demographic characteristics of the patients who died during the study course from AIDS-related diseases versus non-AIDS-related diseases (191 patients).

Demographic and Clinical Characteristics		AIDS	Non-AIDS	*p*-Value
Number of patients ^1^ 191		69 (36.1%)	122 (63.9%)	----
Males		46 (66.6%)	80 (65.5%)	0.86
Females		23 (33.3%)	42 (34.4%)	0.86
Age (years)	Mean age at the time of HIV diagnosis (range)	36.6 ± 13.4 (0.5–73)	43.9 ± 16.4 (0.1–81)	0.003
	Mean age at the time of death (range)	43.9 ± 13.03 (1–73)	52.7 ± 16.2 (18–91)	0.0004
Mean follow-up in years (range)		6.9 ± 5.4 (0.1–21)	8.7 ± 6.05 (0.1–24)	0.2
Risk group	Geographical	54 (78.2%)	89 (72.9%)	0.46
	MSM	11 (15.9%)	17 (13.9%)	0.82
	People who inject drugs	3 (4.3%)	15 (12.4%)	0.08
	MTCT	1 (1.4%)	1 (0.81%)	1.0
CD4 at diagnosis (cells/µL)		222 ± 202	203 ± 173	0.5
VL > 500,000 copies/mL at diagnosis (%)		20 (28.9%)	34 (27.8%)	0.86
VL below detection limit ^2^ (in at least one of the last two tests)		6 (8.7%)	51 (41.8%)	0.0001

^1^ Fifteen patients with unknown cause of death were excluded; ^2^ Viral loads < 400 copies/mL until 2007, viral load < 40 until 2010, viral load < 20 since 2011; MSM = Men who have sex with men, MTCT = Mother to child transmission, VL = viral load.

## Data Availability

The data presented in this study are available on request from the corresponding author due to privacy reasons.

## Abbreviations

HIV—Human Immunodeficiency Virus, HAART—Highly Active Antiretroviral Therapy, AIDS—Acquired Immunodeficiency Syndrome, VL—Viral Load, PLWH—people living with HIV, TB—Tuberculosis, PCP—pneumocystis carinii (Jirovecii) pneumonia, FACS—Fluorescence-Activated Cell Sorting.
